# A relationship between weak attentional control and cognitive distortions, explained by negative affect

**DOI:** 10.1371/journal.pone.0215399

**Published:** 2019-04-18

**Authors:** Robert W. Booth, Dinkar Sharma, Faiqa Dawood, Melis Doğan, Haidy M. A. Emam, Sude S. Gönenç, N. Aslışah Kula, Bengisu Mazıcı, Atakan Saraçyakupoğlu, Asad-Ur-Rehman Shahzad

**Affiliations:** 1 Sabancı University, Istanbul, Turkey; 2 School of Psychology, University of Kent, Canterbury, United Kingdom; Technion Israel Institute of Technology, ISRAEL

## Abstract

People high in negative affect (anxiety or depression) show cognitive distortions, specific thinking errors which contribute to the maintenance of their condition. It is thought that weak attentional control is a risk factor for negative affect and emotional disorders, because weak attentional control exaggerates the expression of attentional bias, another cognitive feature of emotional disorders. We wondered whether weak attentional control might similarly exaggerate the expression of cognitive distortions. In two samples of students from Turkey and the UK, we found that weak attentional control was indeed related to cognitive distortions, but this relationship was explained by both variables’ relationships with negative affect. This suggests that weak attentional control, while related to negative affect, does not necessarily exaggerate all of its cognitive features. There seems to be a limit on the affective consequences of poor attentional control, which may limit its clinical usefulness as a risk factor for emotional disorders.

## Introduction

Disorders related to negative affect, i.e. anxiety and depression, are associated with certain cognitive distortions. In this article, we present evidence that the expression of such cognitive distortions is unrelated to attentional control.

We studied the link between cognitive distortions and attentional control because attentional control has been implicated in the expression of another cognitive feature of emotional disorders and negative affect: attentional bias to threat (e.g.[1]). Studies have reported that attentional bias is more pronounced in high-negative affect individuals who also have poor attentional control: Derryberry and Reed [2] and Reinholdt-Dunne, Mogg, and Bradley [3] found that only participants high in trait anxiety and low in attentional control showed attentional bias to threat; Lonigan and Vasey [4] found the same for negative affectivity; Helzer, Connor-Smith, and Reed [5] and Susa, Benga, Pitică, and Miclea [6] found similar results for trait fearfulness. Booth, Mackintosh and Sharma [7] showed that, in a single group of participants, trait anxiety only predicted attentional bias when attentional control was experimentally impaired (see also [8]). Note that apart from Booth et al. and Reinholdt-Dunne et al., these studies have assessed attentional control using a self-report measure, the Attentional Control Scale [2]. This literature is important because attentional bias may contribute to the aetiology of emotional disorders: basically, it is thought that paying undue attention to potential minor threats tends to increase negative affect, or tends to keep negative affect at a high level [9, 10]. This suggests that anything which exaggerates the expression of attentional bias may also exaggerate negative affect, and is potentially a risk factor for emotional disorders. Theorists have therefore concluded that weak attentional control is such a risk factor [1, 11, 12]; some are even experimenting with training attentional control (or a close correlate) as a potential treatment for emotional disorders [13, 14].

We wanted to know whether the relationship between attentional control and attentional bias would generalise: whether attentional control would influence the expression of other cognitive features of negative affect or emotional disorders. There are two possibilities here: it is implied in the literature (e.g. [1]) that weak attentional control exaggerates attentional bias because attentional bias is essentially a failure of selective attention [15], and weak attentional control will necessarily exaggerate any such failure. If this is true, attentional control should not relate to other cognitive features of negative affect (cf. [7, 16]). Another possibility is that weak attentional control exaggerates attentional bias because attentional control plays a general role in emotion regulation (e.g. [17–19]): people with weak attentional control might show a more exaggerated emotional response to threatening stimuli, leading them to show more attentional bias towards them [20, 21]. This is consistent with evidence that weak attentional control is related to clinical symptoms and behavioural issues in emotional disorders [22–24]. Both explanations imply that weak attentional control is a risk factor for negative affect and emotional disorders, but it would be a more important risk factor under the second account.

In the present studies, we tested whether attentional control was related to the expression of *cognitive distortions*. These are habitual errors in thinking, not necessarily explicitly negative, but characteristic of emotional disorders [25–29]. Much research on cognitive distortions has focused on depression (see e.g. [30]), but they have also been reported in anxiety disorders [31, 32]. Like attentional bias, they are thought to be both a consequence and a cause of negative affect, meaning they may help to maintain emotional disorders [33–37]. Study 1 looked for a relationship between weak attentional control and cognitive distortions in Turkish students in Istanbul; Study 2 examined a larger sample of students studying at a British university. To foreshadow the results, we found no evidence that weak attentional control exaggerates cognitive distortions.

## Study 1

Study 1 was conducted with Turkish students studying at a small private university in Istanbul. We assessed their attentional control using the self-report scale commonly used in the attentional bias literature, their anxiety and depression, and their tendency to exhibit cognitive distortions.

### Method

The study was approved by the Sabancı University Research Ethics Committee (FASS-2018-35), and participants gave their written consent.

#### Participants and procedure

Eighty-two native Turkish speaking students from Sabancı University participated voluntarily. Six were excluded from analyses as they reported a current psychiatric diagnosis other than an anxiety or affective disorder, and another four were excluded for skipping too many questions. The final sample for analysis consisted of 72 students (38 females, 33 males, 1 other, *M*_age_ = 19.47, *SD*_age_ = 0.87). Data were collected in person by the last eight authors, as a class project.

#### Measures

See Table 1 for Cronbach’s α for all variables.

**Table 1:** Bivariate correlations, descriptive statistics and cronbach’s alphas for Study 1.

	2	3	4	5	6	*M*	*SD*	α
1 –BAI	.60 [.42, .73]	-.40 [-.58, -.19]	.47 [.27, .63]	.52 [.33, .67]	.51 [.32, .66]	16.52	10.65	.89
2 –BDI		-.41 [-.59, -.20]	.50 [.30, .66]	.63 [.47, .75]	.59 [.42, .72]	13.70	9.68	.90
3 –ACS			-.41 [-.59, -.20]	-.39 [-.57, -.17]	-.42 [-.59, -.21]	52.60	8.78	.85
4 –Cognitive Distortions (Social)				.85 [.77, .90]	.96 [.94, .98]	39.00	12.75	.88
5 –Cognitive Distortions (Achievement)					.96 [.94, .98]	36.54	13.38	.87
6 –Cognitive Distortions (Total)						75.54	25.11	.94

*p* < .001 for all correlations. *N* = 72. BAI, Beck Anxiety Inventory; BDI, Beck Depression Inventory; ACS, Attentional Control Scale. Square brackets show 95% confidence intervals.

Participants first completed the attentional control scale (ACS; [2], translation by [38]), which asks participants how often 20 statements apply to them. They respond on a 1–4 scale. Items include ‘I have trouble carrying on two conversations at once’ and ‘When I am working hard on something, I still get distracted by events around me.’

They then completed the Beck Anxiety Inventory (BAI; [39], translation by [40]), which asks participants to rate how often have been ‘bothered’ by 21 cognitive and somatic symptoms over the previous month, on a 0–3 scale. Symptoms include ‘nervous’, ‘fear of dying’ and ‘feeling of choking.’

Next, we presented the Beck Depression Inventory-II (BDI; [41], translation by [42]). Participants are presented with 21 groups of four statements, and must choose the one statement which ‘best describes the way [they] have been feeling during the past two weeks.’ Each group of statements measures the severity of a particular cognitive or somatic symptom of depression, including sadness, loss of pleasure, and changes in sleeping pattern.

Finally, they completed the Cognitive Distortions Scale ([26], translation by [27]), which presents descriptions of 10 types of cognitive distortion, along with two examples: one illustrating the distortion in a social context, and one illustrating it in an achievement context. Participants then rate on 1 (Never) to 7 (All the time) scales how often they show each distortion ‘when in social situations (like when you’re with friends, partners or family)’ and ‘when in achievement situations (such as university or work).’ The 10 distortions are mindreading, catastrophising, all-or-nothing thinking, emotional reasoning, labelling, mental filtering, overgeneralisation, personalisation, use of should statements, and minimising or disqualifying the positive. The scale yields scores for the social and achievement contexts, as well as an overall cognitive distortions score (the sum of both context scores). Please see the Supporting Information (Table A in S1 File) for results for the individual distortions.

### Results

First, we checked for a relationship between attentional control and cognitive distortions. Next, we checked whether attentional control moderated anxiety or depression’s relationships with cognitive distortions.

Descriptive statistics and correlations between variables are presented in Table 1. All correlations were significant, *p* < .001, and in the expected directions. Specifically, attentional control correlated negatively with all the other variables; it correlated negatively with cognitive distortions (*r* = -.42), but this correlation was somewhat smaller than anxiety’s and depression’s positive correlations with cognitive distortions (*r*s = .51 and .59).

Given that both attentional control and cognitive distortions correlated with both anxiety and depression, we wanted to check whether attentional control still predicted cognitive distortions independently from the effects of these variables. Total cognitive distortions were regressed on anxiety and depression, with attentional control entered in a second block. The first block model significantly predicted distortions, *R*^2^ = .38, *F* (2, 69) = 21.48, *p* < .001, and the standardised coefficient was significant for both anxiety, β = .25, *p* = .04, and depression, β = .43, *p* < .001. However, adding attentional control did not significantly increase model fit, *R*^2^ change = .02, *F*_change_ (1, 68) = 2.81, *p* = .10. Based on the literature summarised in the Introduction, the lack of a relationship between attentional control and cognitive distortions is surprising, and theoretically important. We studied this further by repeating our analysis as a Bayesian linear regression, using JASP [43] with its default priors. This allowed us to calculate the Bayes factor BF_01_, which indicates how much more likely the observed data are if the tested effect is absent in the population, relative to if the tested effect is present in the population. The BF_01_ for adding attentional control to our model was 1.24, representing weak evidence that attentional control did not have a unique relationship with distortions. The same was true when cognitive distortions in the achievement context replaced total distortions as the outcome, *R*^2^ change = .01, *F*_change_ (1, 68) = 1.62, *p* = .21, BF_01_ = 2.25; when distortions in the social context as the outcome, attentional control marginally increased model fit, *R*^2^ change = .03, *F*_change_ (1, 68) = 3.51, *p* = .07, BF_01_ = 0.81, but upon closer inspection this result was due to a strong correlation between attentional control and one particular distortion, the use of should statements (see Supporting Information, Table A in S1 File). When this one distortion was removed, attentional control did not improve model fit with distortions in the social context as the outcome *R*^2^ change = .02, *F*_change_ (1, 68) = 2.35, *p* = .13, BF_01_ = 1.38. This suggests that, with the exception of should statements in the social context, the relationship between attentional control and cognitive distortions is accounted for by anxiety and depression.

We next checked to see whether attentional control moderated anxiety or depression’s relationships with cognitive distortions: as described in the Introduction, attentional control tends to moderate negative affect’s relationship with attentional bias. Total distortions were regressed on mean-centred attentional control and anxiety, with their interaction term entered in a second block. This interaction term did not improve model fit, *R*^2^ change = .0002, *F*_change_ (1, 68) = 0.02, *p* = .88, BF_01_ = 3.91. We then checked whether attentional control moderated depression’s relationship with distortions; it did not, *R*^2^ change = .001, *F*_change_ (1, 68) = 0.15, *p* = .70, BF_01_ = 4.11. Finally, we repeated both moderation analyses with both social and achievement distortions as outcomes, no significant interactions emerged (all *p*s > .22). These results suggest that attentional control does not moderate the relationship between negative affect (anxiety or depression) and cognitive distortions.

### Discussion

Attentional control was related to the expression of cognitive distortions, but this relationship became nonsignificant when anxiety and depression were controlled, suggesting the relationship was explained by negative affect. Attentional control also did not moderate anxiety or depression’s relationships with cognitive distortions. These findings suggest that weak attentional control, while related to negative affect, does not necessarily exaggerate the expression of all negative affect’s cognitive features. There seems to be some limit on the psychopathological sequelae of poor attentional control, which may limit its clinical usefulness as a risk factor for emotional disorders.

The Bayes factors for the moderation analyses above indicate quite convincing evidence that attentional control does not moderate negative affect’s relationship with cognitive distortions. However, the Bayes factors for the relationship between attentional control and distortions when negative affect is controlled do not indicate convincing evidence. In other words, Study 1 cannot completely rule out the possibility that there may be a unique relationship between attentional control and cognitive distortions. This is probably an issue of sample size; in Study 2 we were able to collect a much larger sample, and found more convincing evidence that no such unique relationship exists.

One complicating issue is that attentional control seemed to show a unique relationship with one particular distortion, the use of should statements in the social context. This is due to an unusually strong correlation between attentional control and this distortion (*r* = -.40), which is rather stronger than the correlation between this distortion and depression (*r* = .28). This finding was not replicated in Study 2’s UK sample, reported below; so it is unclear whether this finding reflects a simple Type I error, or whether Turkish students are more likely to show such a relationship than are UK students. Özdel et al. [27] report correlations between individual cognitive distortions, measured with the same instrument, and BDI and trait anxiety scores for a non-clinical Turkish sample: their correlations for should statements are eerily close to ours, although their correlations for the other nine distortions are not. This result is particularly puzzling given that the Cognitive Distortions Scale’s authors reported a unitary structure for the instrument, and recommended using the total score rather than scores for individual distortions [26]; this together with the high internal consistency of the instrument in this sample (Table 1) suggests that all the distortions should show similar relationships with our predictors. At this stage we can do little except to note this issue for future investigation.

## Study 2

Study 2 sought to replicate the results of Study 1 in a larger sample, this time from the UK. This study included an extra measure of anxiety, the trait scale of the state-trait anxiety inventory (STAI, [44]). Although designed as an anxiety measure, the STAI has proven to be sensitive to generalised negative affect [45, 46]. Since we suspected that the attentional control-cognitive distortions link might be accounted for by general negative affect rather than anxiety or depression specifically, we wanted to measure such negative affect as completely as possible. This scale measures anxiety/affect as a long-term trait, unlike the Beck Anxiety Inventory which assesses anxiety symptoms over the previous month, so to increase comparability of the two scales we also switched to the trait version of the BAI (the ‘BAIT’). However, results from the two scales turned out to be very similar.

### Method

The University of Kent’s Psychology Ethics Committee approved the study (201815180106374922), and participants gave their consent electronically before beginning the study.

#### Participants and procedure

Two hundred and nine undergraduates from the University of Kent participated for course credit. Ten participants were excluded from analyses as they reported a current psychiatric diagnosis other than an anxiety or affective disorder (excluding all participants with a current psychiatric diagnosis does not alter the results), or chose not to disclose their diagnoses. The final sample for analysis therefore included 199 students (167 females, 30 males, 2 others, *M*_age_ = 19.59, *SD*_age_ = 3.02). Participants were told the study was about “thinking styles, and which personal characteristics affect habits of thinking.” They completed the scales online.

#### Measures

See Table 2 for internal consistency and descriptive statistics for all measures.

**Table 2:** Bivariate Correlations, Descriptive Statistics and Cronbach’s Alphas for Study 2.

	2	3	4	5	6	7	8	*M*	*SD*	α
1 –STAI-T	.63**[.54; .71]	.74**[.67; .80]	-.39**[-.50; -.27]	-.17*[-.30; -.03]	.57**[.47; .66]	.51**[.40; .61]	.57**[.47; .66]	46.23	8.54	.87
2 –BAIT		.62**[.53; .70]	-.38**[-.49; -.25]	-.18*[-.31; .04]	.40**[.28; .51]	.40**[.28; .51]	.42**[.30; .53]	13.86	10.52	.93
3 –BDI			-.38**[-.49; -.25]	-.16*[-.29; -.02]	.53**[.42; .62]	.49**[.38; .59]	.53**[.42; .62]	15.78	11.91	.91
4 –ACS				.29**[.16; .41]	-.28**[-.40; -.15]	-.25**[-.38; .12]	-.28**[-.40; -.15]	49.38	8.91	.84
5 –MCSD					-.18*[-.31; -.04]	.15*[.01; .28]	-.17*[-.30; -.03]	48.96	4.24	.64
6 –Cognitive Distortions (Social)						.83** [.78; .87]	.96**[95.; .97]	42.32	11.36	.87
7 –Cognitive Distortions (Achievement)							.96**[.95; .97]	42.10	11.36	.87
8 –Cognitive Distortions (Total)								84.24	21.74	.93

*N* = 199. STAI-T, State-Trait Anxiety Inventory, trait subscale; BAIT, Beck Anxiety Inventory, trait version; BDI, Beck Depression Inventory; ACS, Attentional Control Scale; MCSD, Marlowe-Crowne Social Desirability. Square brackets show 95% confidence intervals.

* *p* < .05

** *p* < .001

Participants first completed the State-Trait Anxiety Inventory, trait subscale [44], which includes 20 items assessing how participants ‘generally, in [their] life’. Participants respond on a 1–4 scale. Items include ‘I worry too much over something that really doesn’t matter’ and ‘I have disturbing thoughts.’

Participants then completed the BAIT, which is a version of the BAI which is designed to measure anxiety as a trait, rather than over a specific timescale [47]. The items are identical to the standard BAI, but the instructions ask the respondent to rate how often each symptom affects them in their lives in general. They then completed the ACS, BDI, and Cognitive Distortions Scale.

Finally, participants completed the Marlowe-Crowne social desirability scale (MCSD; [48]). This presents 33 statements such as ‘I have never intensely disliked anyone’ and ‘I am always willing to admit it when I make a mistake’, and asks participants whether each statement is true of them. We often include this scale in our studies because participants may not be honest about their anxiety or depression. This turned out to be unnecessary for this study, and the scale showed unacceptable psychometric performance. We therefore do not mention this scale again, but we do include it in Table 2.

### Results

Again, we first checked for a relationship between attentional control and cognitive distortions, and then whether attentional control moderated anxiety’s or depressions’ relationship with cognitive distortions.

Correlations are presented in Table 2; analyses of individual cognitive distortions are presented in the Supporting Information (Table B in S1 File). As expected, trait anxiety (both measures) and depression significantly predicted total cognitive distortions, and cognitive distortions in both the social and achievement contexts. More importantly, attentional control negatively predicted cognitive distortions in the social context, in the achievement context, and in total.

Again, we investigated the relationship between attentional control and cognitive distortions using hierarchical regression. Total cognitive distortions were regressed on BAIT and BDI scores in a first block, and attentional control was added in a second block. The first block model significantly predicted cognitive distortions, *R* = .54, *R*^2^ = .30, *F* (2, 196) = 41.27, *p* < .001; the standardised coefficient was marginal for BAIT (β = .15, *p* = .057) and significant for BDI (β = .44, *p* < .001). However, adding attentional control did not improve model fit, *R*^2^ change = .003, *F*_change_ (1, 195) = 0.92, *p* = .34, indicating that the relationship between attentional control and cognitive distortions is accounted for by anxiety and depression: there is no unique relationship between these variables. This analysis was repeated as a Bayesian regression as before, comparing the full model to a null model including only BAIT and BDI. The BF_01_ for adding attentional control was 4.14, indicating good evidence that there truly is no unique relationship between attentional control and cognitive distortions. The hierarchical regression analysis was repeated for cognitive distortions in both the social and achievement contexts, and then all three analyses were repeated with STAI trait anxiety in the model in place of the BAIT. Attentional control did not improve model fit in any of these analyses (all *R*^2^ changes < .005, all *p*s > .25).

Again, moderated regression was used to check for any interaction between trait anxiety and attentional control on total distortions. The interaction term did not significantly improve model fit, *R*^2^ change = .006, *F*_change_ (1, 195) = 1.34, *p* = .25. Bayesian linear regression showed that the evidence for the interaction’s being absent was somewhat stronger than the evidence for it being present, BF_01_ = 2.81. These analyses were then repeated with STAI trait anxiety in the model instead of the BAIT; again, adding the interaction term did not improve model fit, *R*^2^ change = .004, *F*_change_ (1, 195) = 1.12, *p* = .29, and the evidence for the interaction’s absence was stronger than the evidence for its presence, BF_01_ = 3.97. There was a significant interaction between depression and attentional control on total distortions, *R*^2^ change = .02, *F*_change_ (1, 195) = 6.76, *p* = .01, but this did not resemble the interaction of negative affect and attentional control on attentional bias reported in the literature: simple slopes calculated for low (mean– 1 *SD*), medium (mean), and high (mean + 1 *SD*) levels of depression revealed that attentional control only predicted distortions when depression was low (β = -.27, *p* = .01), and not when depression was medium (β = -.11, *p* = .08) or high (β = .05, *p* = .57), where distortions were more uniformly high. Essentially, attentional control’s relationship with cognitive distortions was only visible when depression was low; this simply shows that attentional control’s relationship with distortions was rather weaker than depression’s relationship with distortions. It was not the case that distortions were strongest when depression was high and control was low, as found in the attentional bias studies reviewed in the Introduction.

### Discussion

Again, attentional control showed a significant relationship with cognitive distortions; however, this relationship seems to be reliably and wholly accounted for by negative affect. The correlations between attentional control and STAI trait anxiety, BAIT trait anxiety, and depression were all very similar, suggesting that it is negative affect in general, not anxiety or depression specifically, which accounts for the relationship between attentional control and cognitive distortions.

Unlike Study 1, this study found evidence for an interaction between depression and attentional control on cognitive distortions. However, this interaction was very unlike the interaction reported in the literature between attentional control and negative affect on attentional bias to threat. Instead, we found that attentional control’s relationship with distortions was only visible when depression was low; this is not consistent with the idea that low attentional control increases the expression of affect-related cognitive distortions. Rather, both these studies suggest that weak attentional control does not necessarily exaggerate all cognitive features of negative affect.

## General discussion

In two studies, attentional control significantly predicted cognitive distortions, a key symptom–and presumably, a key cause–of negative affect and emotional disorders. However, more careful analyses showed that this relationship was essentially an artefact, resulting from the fact that both control and distortions correlate with negative affect (i.e., with anxiety and depression).

This could happen for two reasons. One possibility is that both attentional control and cognitive distortions are both independently related to negative affect, but not to each other. The other possibility is that people high in negative affect might have a response bias, so that they tend to respond in a negative way on any scale concerning their own performance or abilities, including both the ACS and the cognitive distortions scale; this could be considered a kind of common method bias (see [49]; but see below). In either case, this would mean that the link between ACS-measured attentional control and some cognitive symptoms of emotional disorders (e.g. attentional bias) is limited, and does not generalise to cognitive distortions.

It is not clear why attentional control plays a role in some affect-related cognitive biases [4, 16] and in emotion regulation [18, 19], but no role in cognitive distortions. It is certainly not the case that attentional control is only related to attention-based emotional behaviours [16, 17]. Perhaps part of the reason is that cognitive distortions are habits of thought, rather than direct responses to emotions or emotional situations; they are not always intrinsically positive or negative, but can be unvalenced thoughts which tend to result from negative affect and emotional disorders. In other words, they are habits which correlate with variations in trait affect, they do not reflect state variations in affect or mood. The relevance of these habits of thought for affect is often not obvious to people with emotional disorders–indeed, cognitive behavioural therapy involves helping the client to identify these distortions, and understand the harm they cause [33]–so they may be less amenable to emotional regulation processes, including attentional control.

Importantly, this does not devalue previous studies on attentional control and attentional bias, neither does it invalidate their conclusions. Attentional bias is believed to be important for the causation and maintenance of high anxiety, so any factor which exaggerates this bias is necessarily a risk factor for anxiety disorders. Indeed, we found consistent negative associations of about *r* = -.40 between attentional control and all our psychopathology measures (see also [50, 51]), which may indicate that individuals with weaker attentional control will find it harder to shift their attention away from negatively valenced information in their environments [1], or that attentional control plays a role in emotion regulation [19]. However, these findings show that there is some limit to attentional control’s role in the expression of cognitive features of negative affect and emotional disorders. Cognitive distortions are particularly crucial here because they play a key causative role in anxiety and depression, and are a key target of cognitive behavioural therapy, which is very effective at remediating them [52]. These results suggest that, if training attentional control can be used to treat these emotional disorders [13, 14], it could complement rather than replace traditional psychotherapy.

One obvious limitation of these studies is that they relied on a self-report measure of attentional control. There is mixed evidence concerning the validity of the ACS [51, 53], with some authors arguing that the scale measures beliefs about attentional control ability rather than ability itself [54], which could mean that people high in negative affect respond negatively to ACS questions regardless of their real abilities. Certainly it remains possible that attentional control, when measured using a behavioural test, could have a unique relationship with cognitive distortions, and future studies should check this. However, we suggest this is unlikely: Booth and Sharma (manuscript under review) recently found that ACS scores predicted another cognitive feature of emotional disorders–the tendency to feel that negative events are more likely than positive events [55]–when trait anxiety, depression, and emotion regulation were simultaneously controlled. This result suggests that some unique relationship exists between the scale and cognitive biases, which cannot be explained by a negative response bias in people high in negative affect. And yet, no such unique relationship with between attentional control and cognitive distortion appeared in the present studies, despite Study 2 having .80 power to detect a negative relationship as small as *f*^2^ = .03 ([56], calculated using G*Power); an effect smaller that this may be of minimal clinical relevance. Besides, the validity of the attentional control scale is actually not so relevant to our argument: the claim that weak attentional control is a risk factor for anxiety and depression [1, 11] is based largely on studies showing this scale relates to the expression of attentional bias [2, 4, 6], and the present studies have shown that, whatever may cause this relationship, it has no influence on cognitive distortions.

To conclude, these studies demonstrate that there are limits to poor attentional control’s exaggeration of negative affect and its cognitive features. The next step is to investigate exactly where this limit lies, and to investigate how useful attentional control is for predicting and remediating emotional disorders.

## Supporting information

S1 File Bivariate correlations and descriptive statistics for individual cognitive distortions. (PDF)
