# Supplementary material for: A relationship between weak attentional control and cognitive distortions, explained by negative affect
Source: PLoS One. 2019 Apr 18;14(4):e0215399. doi: 10.1371/journal.pone.0215399 (PMC6472758; doi:10.1371/journal.pone.0215399)
Supplement: S1 File — (PDF) [file pone.0215399.s001.pdf]

S1 File. Bivariate correlations and descriptive statistics for individual cognitive distortions.

Table A. Bivariate correlations and descriptive statistics for individual cognitive distortions, Study 1. Each distortion is measured with a single item.

	Social Context										Achievement Context									
	1	2	3	4	5	6	7	8	9	10	1	2	3	4	5	6	7	8	9	10
BAI	.38**	.50***	.23	.22	.32**	.40***	.45***	.21	.20	.32**	.38***	.57***	.25*	.39***	.37**	.35**	.50***	.21	.26*	.35**
BDI	.30*	.57***	.25*	.17	.30*	.28*	.41***	.33**	.28*	.52***	.28*	.56***	.39***	.45***	.44***	.37**	.66***	.38***	.34**	.49***
ACS	-.19	-.33**	-.28*	-.20	-.34**	-.24*	-.24*	-.22	-.40***	-.38***	-.16	-.51***	-.14	-.36**	-.38***	-.03	-.38***	-.20	-.25*	-.32**
M	4.90	3.92	3.69	4.17	3.71	3.76	3.53	3.71	3.92	3.71	4.28	3.76	3.83	3.69	3.38	3.64	3.31	3.33	4.04	3.29
SD	1.74	1.98	1.90	1.77	1.94	1.68	1.81	1.90	1.81	1.95	1.73	1.97	2.13	1.81	1.95	1.67	1.98	1.91	1.91	1.97

Note. * $p < .05$; ** $p < .01$; *** $p < .001$. $N = 72$. BAI, Beck Anxiety Inventory. BDI, Beck Depression Inventory. ACS, Attentional Control Scale. 1, mindreading; 2, catastrophizing; 3, all-or-nothing thinking; 4, emotional reasoning; 5, labelling; 6, mental filtering; 7, overgeneralisation; 8, personalisation; 9, should statements; 10, minimising or disqualifying the positive.

Table B. Bivariate correlations and descriptive statistics for individual cognitive distortions, Study 2.

	Social Context										Achievement Context									
	1	2	3	4	5	6	7	8	9	10	1	2	3	4	5	6	7	8	9	10
STAI-T	$.34^{***}$	$.40^{***}$	$.21^{**}$	$.49^{***}$	$.45^{***}$	$.39^{***}$	$.42^{***}$	$.41^{***}$	$.30^{***}$	$.47^{***}$	$.24^{***}$	$.34^{***}$	$.34^{***}$	$.38^{***}$	$.38^{***}$	$.36^{***}$	$.37^{***}$	$.33^{***}$	$.31^{***}$	$.41^{***}$
BAI	$.28^{***}$	$.27^{***}$	.13	$.29^{***}$	$.33^{***}$	$.30^{***}$	$.27^{***}$	$.37^{***}$	$.16^{*}$	$.32^{***}$	$.23^{**}$	$.20^{**}$	$.20^{**}$	$.31^{***}$	$.30^{***}$	$.29^{***}$	$.28^{***}$	$.36^{***}$	$.24^{***}$	$.31^{***}$
BDI	$.32^{***}$	$.33^{***}$	$.17^{*}$	$.46^{***}$	$.40^{***}$	$.35^{***}$	$.45^{***}$	$.39^{***}$	$.26^{***}$	$.43^{***}$	$.21^{**}$	$.32^{***}$	$.32^{***}$	$.30^{***}$	$.36^{***}$	$.32^{***}$	$.43^{***}$	$.33^{***}$	$.32^{***}$	$.41^{***}$
ACS	$-.18^{*}$	$-.19^{**}$	-.13	$-.32^{***}$	$-.20^{**}$	$-.19^{**}$	$-.27^{***}$	$-.20^{**}$	-.04	$-.21^{**}$	-.11	$-.21^{**}$	-.07	$-.22^{**}$	$-.19^{**}$	-.13	$-.28^{***}$	-.16	-.12	$-.19^{**}$
M	5.06	4.27	3.30	4.74	4.09	4.64	4.10	3.89	4.09	4.14	4.47	4.78	4.07	4.12	4.27	4.42	4.29	3.50	4.33	3.85
SD	1.56	1.69	1.59	1.62	1.72	1.66	1.68	1.60	1.68	1.90	1.60	1.66	1.77	1.59	1.76	1.65	1.68	1.59	1.76	1.76

Note. * $p < .05$; $^{**}p < .01$; *** $p < .001$. $N = 199$. STAI-T, State-Trait Anxiety Inventory, trait subscale. BAI, Beck Anxiety Inventory. BDI, Beck Depression Inventory. ACS, Attentional Control Scale. 1, mindreading; 2, catastrophizing; 3, all-or-nothing thinking; 4, emotional reasoning; 5, labelling; 6, mental filtering; 7, overgeneralisation; 8, personalisation; 9, should statements; 10, minimising or disqualifying the positive.
